# Restoration of normal blood flow in atherosclerotic arteries promotes plaque stabilization

**DOI:** 10.1016/j.isci.2023.106760

**Published:** 2023-04-27

**Authors:** Morgan A. Schake, Ian S. McCue, Evan T. Curtis, Thomas J. Ripperda, Samuel Harvey, Bryan T. Hackfort, Anna Fitzwater, Yiannis S. Chatzizisis, Forrest M. Kievit, Ryan M. Pedrigi

**Affiliations:** 1 Department of Mechanical and Materials Engineering, University of Nebraska-Lincoln, Lincoln, NE 68588, USA; 2 Department of Biological Systems Engineering, University of Nebraska-Lincoln, Lincoln, NE 68583, USA; 3 Department of Cellular and Integrative Physiology, University of Nebraska Medical Center, Omaha, NE 68198, USA; 4 Institutional Animal Care Program, University of Nebraska-Lincoln, Lincoln, NE 68583, USA; 5 Division of Cardiovascular Medicine, University of Nebraska Medical Center, Omaha, NE 68198, USA; 6 Nebraska Center for Integrated Biomolecular Communication, University of Nebraska-Lincoln, Lincoln, NE 68588, USA

**Keywords:** Cardiovascular medicine, Vascular remodeling

## Abstract

Blood flow is a key regulator of atherosclerosis. Disturbed blood flow promotes atherosclerotic plaque development, whereas normal blood flow protects against plaque development. We hypothesized that normal blood flow is also therapeutic, if it were able to be restored within atherosclerotic arteries. Apolipoprotein E-deficient (ApoE^−/−^) mice were initially instrumented with a blood flow-modifying cuff to induce plaque development and then five weeks later the cuff was removed to allow restoration of normal blood flow. Plaques in decuffed mice exhibited compositional changes that indicated increased stability compared to plaques in mice with the cuff maintained. The therapeutic benefit of decuffing was comparable to atorvastatin and the combination had an additive effect. In addition, decuffing allowed restoration of lumen area, blood velocity, and wall shear stress to near baseline values, indicating restoration of normal blood flow. Our findings demonstrate that the mechanical effects of normal blood flow on atherosclerotic plaques promote stabilization.

## Highlights

• Removal of a blood flow modifying cuff restores normal blood flow
• Restored normal blood flow in atherosclerotic arteries causes plaque stabilization
• The combination of restored normal flow and atorvastatin causes plaque regression

## Graphical abstract

**Figure:**
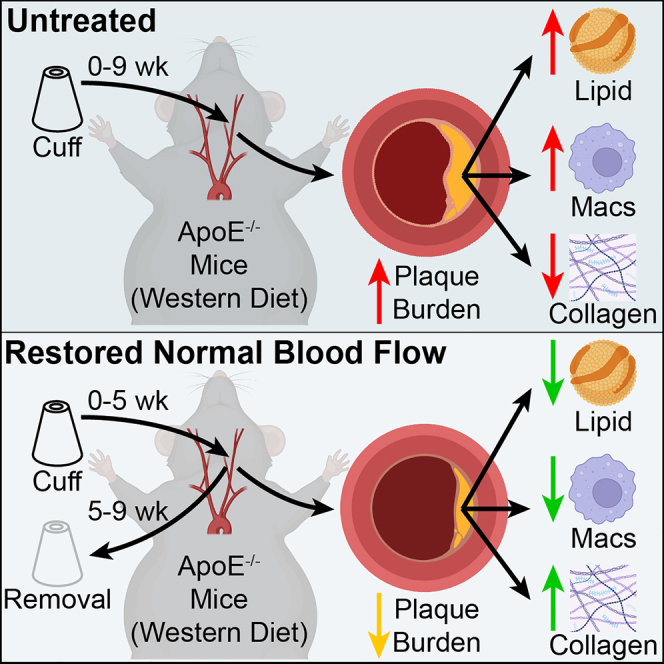

## Introduction

Blood flow within an artery is central to both the initiation and progression of atherosclerotic plaques.^1^^,^^2^ The interaction of blood flow with arteries of different geometries induces different mechanical shear stresses onto the endothelium of the inner artery wall.^3^ The shear stress condition plays a key role in determining the susceptibility of an arterial segment to chronic inflammation and the accumulation of cholesterol-containing low-density lipoprotein to form plaques.^4^ Arterial segments with bifurcations or high curvature that contain so-called disturbed blood flow and associated shear stresses promote plaque development.^2^^,^^4^ On the other hand, relatively straight arterial segments contain normal blood flow or unidirectional flow at a normal magnitude that protects against plaque development.^3^^,^^5^ Although these relationships are well accepted, our understanding remains incomplete. It is still unknown what effect normal blood flow has on existing plaques, if it were able to be restored within atherosclerotic arteries.

Studies investigating the importance of blood flow in atherosclerosis have focused on associations between wall shear stress (WSS) metrics of disturbed flow and plaque features. They have consistently shown that plaques exposed to low WSS are associated with significantly increased burden, necrotic core area, lipids, and macrophages, as well as reduced fibrous cap thickness.^6^^,^^7^^,^^8^ Pig studies have shown broadly similar results, demonstrating that arterial segments with low WSS are associated with the vulnerable plaque phenotype, thin cap fibroatheroma (TCFA), and numerous plaque features, including: increased plaque size, lipids, macrophages and other inflammatory markers, as well as decreased collagen and fibrous cap thickness.^9^^,^^10^ In addition to low WSS, studies in pigs and humans have also demonstrated that multidirectional WSS and the combination of low/multidirectional WSS is associated with many of these advanced plaque features.^11^^,^^12^

An important limitation of these studies is that they do not demonstrate a causal relationship between blood flow and plaque progression. Our study in pigs demonstrated that implantation of stenotic stents in the coronary arteries induced disturbed blood flow and caused the development of TCFA in regions of persistently low WSS.^1^ This pig model was motivated by a similar ApoE^−/−^ mouse model wherein disturbed blood flow is induced by placement of a tapered cuff around one of the carotid arteries.^13^ The arterial segments upstream and downstream of the cuff experience low WSS and low/multidirectional WSS, respectively, which causes plaque development in each region.^14^ The upstream plaque exhibits an unstable phenotype that is lipid-rich with increased macrophages and inflammatory mediators and decreased collagen compared to the more stable downstream plaque.^13^^,^^14^^,^^15^^,^^16^ In line with the known atheroprotective effects of normal and high unidirectional WSS,^3^ neither the arterial segment within the tapered cuff nor the contralateral control artery develop plaques, despite the presence of severe hypercholesterolemia.^13^

To date, studies of the relationship between blood flow and atherosclerosis have entirely focused on the deleterious effects of disturbed flow. This single direction of inquiry has greatly improved our ability to understand and predict atherosclerosis development. Yet, there remains a need for complementary studies investigating the potentially beneficial effects of normal blood flow that could lead to the development of novel therapeutics for atherosclerosis. The atheroprotective nature of normal blood flow suggests that it could be therapeutic, but this has not been directly evaluated. Therefore, in this study, we investigated the hypothesis that restoration of normal blood flow within atherosclerotic arteries promotes plaque stabilization. Our ApoE^−/−^ mouse model provided the ideal platform for this investigation by allowing cuff placement to induce atherosclerosis followed by cuff removal or decuffing to restore normal blood flow. Flow restoration as a result of decuffing was demonstrated using serial *in vivo* magnetic resonance (MR) imaging and Doppler ultrasound. CFD modeling demonstrated restored normal WSS conditions. Histology was performed to evaluate plaque burden, lipids, macrophages, and collagen. We found that plaques in decuffed mice, particularly those in the unstable plaque region, had significantly improved stability compared to those in the untreated cuffed mice. The extent of this plaque stabilization in decuffed mice was comparable to cuffed mice treated with atorvastatin and the combination of the two had an additive therapeutic effect.

## Results

### Decuffing promotes plaque stabilization comparable to treatment with atorvastatin and the combination has an additive therapeutic effect

**Figure 1:**
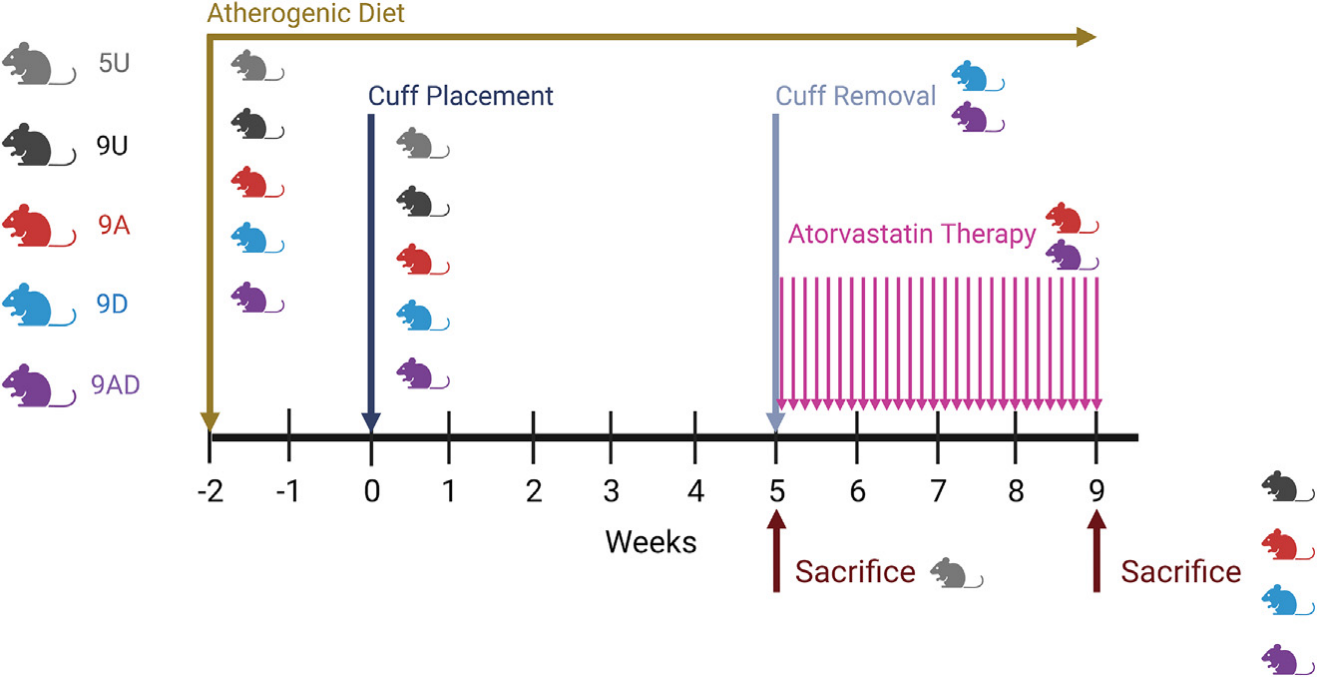
Diagram of the study experimental design The experimental groups are: 5-Untreated (5U), 9-Untreated (9U), 9-Atorvastatin (9A), 9-Decuffed (9D), and 9-Atorvastatin-Decuffed (9AD).

To evaluate the therapeutic benefit of decuffing alone and in combination with atorvastatin, mice were randomly assigned to one of the following five experimental groups: (1) Untreated with the cuff maintained for five weeks (5U), (2) untreated with the cuff maintained for nine weeks (9U), (3) treated with atorvastatin daily after five weeks of cuff placement and maintained with the cuff for an additional four weeks (9A), (4) treated with decuffing (to restore normal blood flow) after five weeks of cuff placement and maintained without the cuff for an additional four weeks (9D), and (5) treated with the combination of atorvastatin plus decuffing (9AD). All treatments commenced five weeks after initial cuff placement (denoted week 5) when plaques are at an intermediate stage of development.^13^^,^^15^ A summary of the experimental protocol is given in Figure 1.

**Figure 2:**
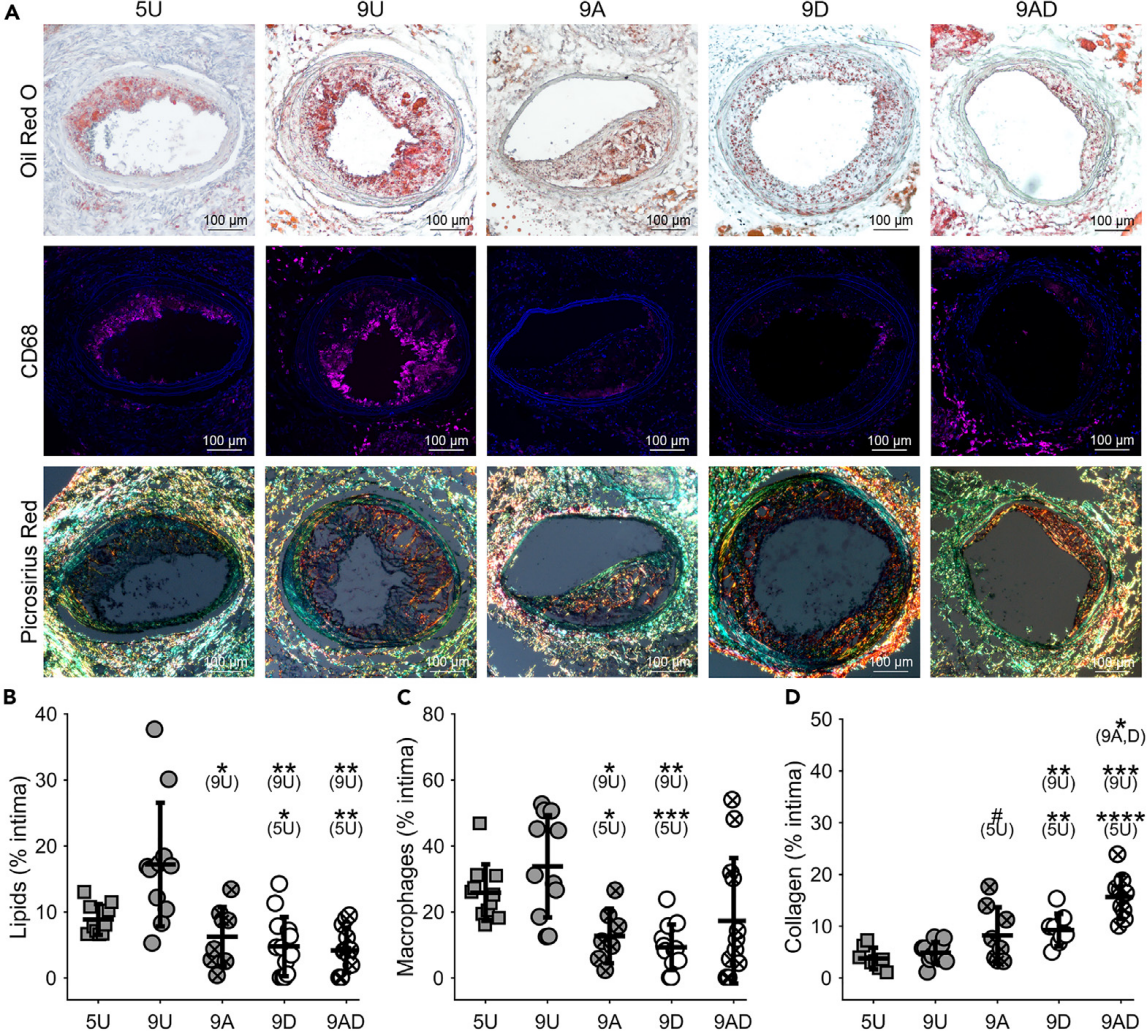
Decuffing to restore normal blood flow promotes increased stability of plaques in the unstable (upstream) plaque region comparable to treatment with atorvastatin and the combination induced an additive therapeutic effect (A) Representative histology sections stained for oil red O (lipids), CD68 (macrophages), and picrosirius red (collagen) for the 5-Untreated (5U), 9-Untreated (9U), 9-Atorvastatin (9A), 9-Decuffed (9D), and 9-Atorvastatin-Decuffed (9AD) groups. Scale bars are 100 μm. (B–D) Boxplots of (B) lipids (*n* = 8–13 mice per group), (C) macrophages (*n* = 7–12 mice per group), and (D) collagen (*n* = 8–11 mice per group) content. Bars represent mean ± SD. ∗Indicates statistically significant difference for comparison given parenthetically, wherein ^#^p<0.1, ∗p<0.05, ∗∗p<0.01, ∗∗∗p<0.001, and ∗∗∗∗p<0.0001. See also Table S1.

Placement of a blood flow-modifying cuff around the left carotid artery of ApoE^−/−^ mice induced the development of plaques in both the unstable (upstream) and stable (downstream) plaque regions. In the unstable plaque region, decuffed mice exhibited significant changes in plaque composition (Figure 2A). In this treatment group (9D) compared to untreated groups (5U and 9U), plaque lipid content was lower (4.8 ± 4.5% versus 8.9 ± 2.3% (p = 0.04) and 17.2 ± 9.4% (p = 0.005), respectively) (Figure 2B), macrophage (CD68^+^ cells) content was lower (9.3 ± 6.9% versus 25.9 ± 8.5% (p = 0.0002) and 33.8 ± 15.5% (p = 0.001), respectively) (Figure 2C), and collagen content was higher (9.3 ± 3.1% versus 3.8 ± 2.1% (p = 0.004) and 4.9 ± 2.0% (p = 0.005), respectively) (Figure 2D). Similar results were seen in mice treated with atorvastatin (9A) and there were no significant differences in plaque composition between the 9A and 9D groups. The mice treated with the combination of atorvastatin plus decuffing (9AD) exhibited similar plaque lipid and macrophage contents, but higher collagen content compared to the individual treatment groups (15.7 ± 4.2% versus 8.2 ± 5.4% (p = 0.01) and 9.3 ± 3.1% (p = 0.008) for the 9A and 9D groups, respectively) (Figures 2B–2D).

**Figure 3:**
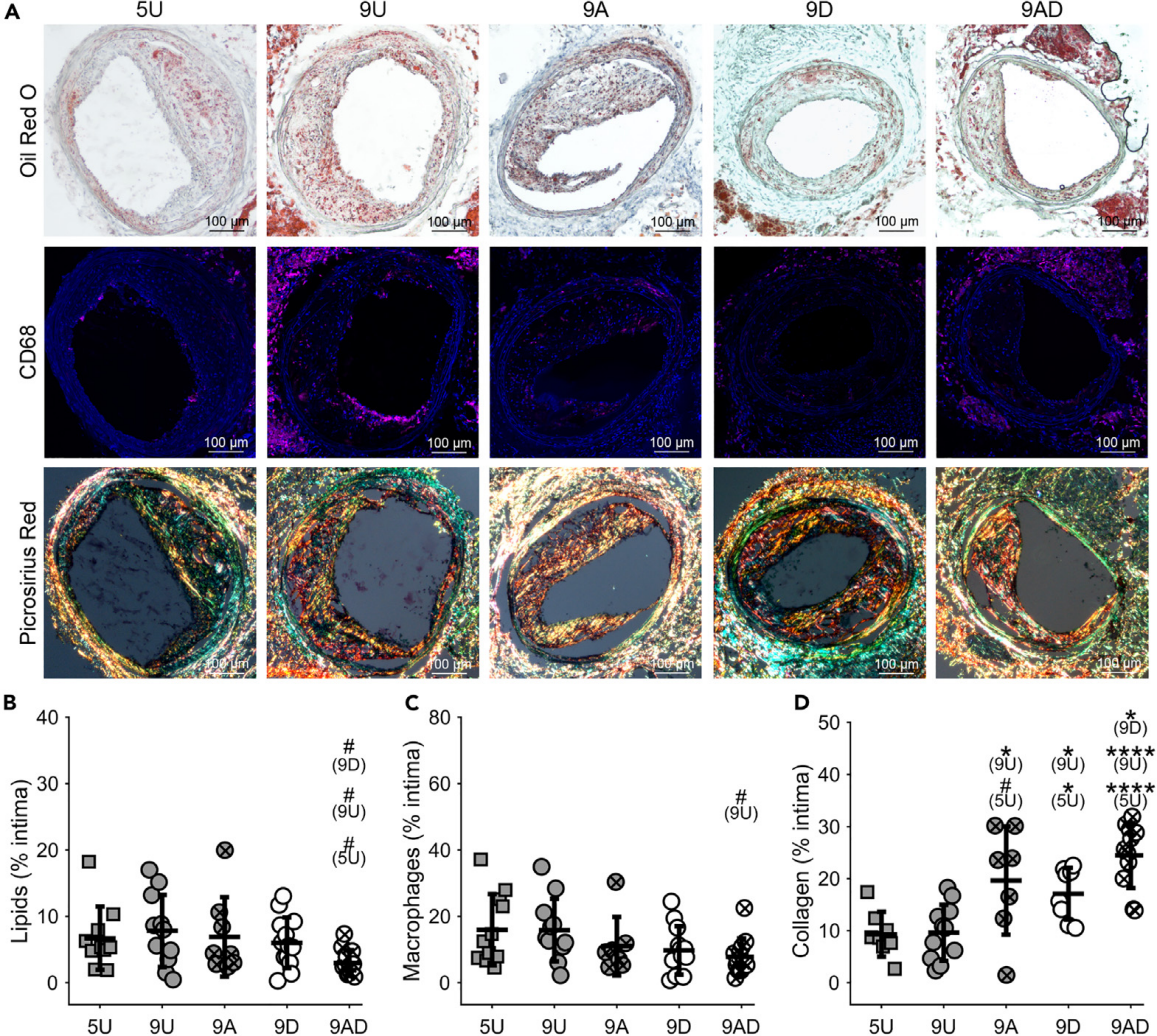
Decuffing to restore normal blood flow promotes increased stability of plaques in the stable (downstream) plaque region comparable to treatment with atorvastatin and the combination induced an additive therapeutic effect (A) Representative histology sections stained for oil red O (lipids), CD68 (macrophages), and picrosirius red (collagen) for the 5-Untreated (5U), 9-Untreated (9U), 9-Atorvastatin (9A), 9-Decuffed (9D), and 9-Atorvastatin-Decuffed (9AD) groups. Scale bars are 100 μm. (B–D) Boxplots of (B) lipids (*n* = 8–13 mice per group), (C) macrophages (*n* = 7–12 mice per group), and (D) collagen (*n* = 7–11 mice per group) content. Bars represent mean ± SD. ∗Indicates statistically significant difference for comparison given parenthetically, wherein ^#^p<0.1, ∗p<0.05, and ∗∗∗∗p<0.0001. See also Table S2.

In the stable plaque region, the 9AD group also exhibited the most substantial changes in plaque composition (Figure 3A). Here, plaque lipid content was similar between experimental groups, except the 9AD group, which exhibited a lower value (3.0 ± 1.9%) compared to the 5U (6.7 ± 4.8%, p = 0.07), 9U (7.8 ± 5.4%, p = 0.07), and 9D (6.0 ± 3.8%, p = 0.07) groups (Figure 3B), though the adjusted p values were just above the threshold for significance. The 9AD group also exhibited the lowest plaque macrophage content compared to the other groups, though none of the comparisons reached significance (Figure 3C). However, collagen content was significantly higher in all treatment groups compared to the untreated groups (except 9A versus 5U) and the 9AD group had the highest plaque collagen content of 24.5 ± 6.2%. This value was significantly higher than the 5U (9.3 ± 4.3%, p<0.0001), 9U (9.6 ± 5.4%, p<0.0001), and 9D (17.1 ± 5.0%, p = 0.03) groups (Figure 3D).

**Figure 4:**
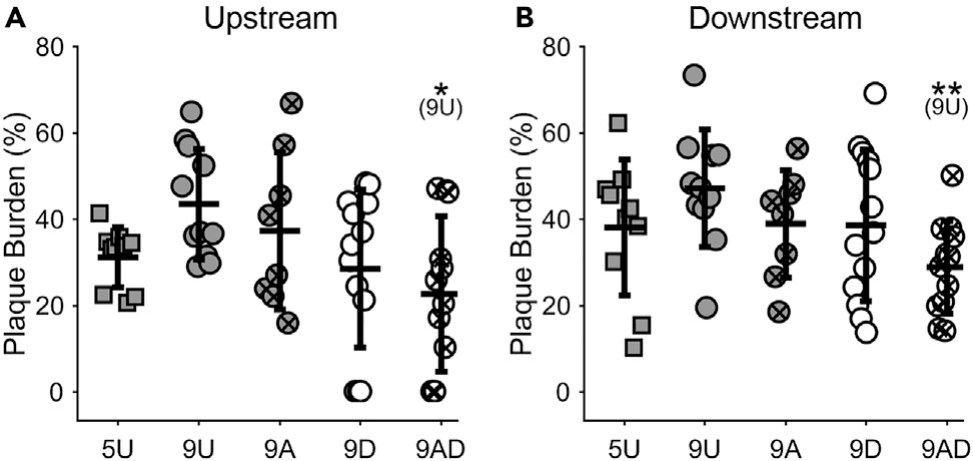
The combination of atorvastatin and decuffing reduces plaque burden (A and B) Plaque burden in the (A) upstream (unstable plaque) and (B) downstream (stable plaque) regions of the instrumented artery (*n* = 8–13 mice per group). Experimental groups are the 5-Untreated (5U), 9-Untreated (9U), 9-Atorvastatin (9A), 9-Decuffed (9D), and 9-Atorvastatin-Decuffed (9AD). Bars represent mean ± SD. ∗Indicates statistically significant difference for comparison given parenthetically, wherein ∗p<0.05 and ∗∗p<0.01. See also Table S3.

In addition to altered plaque composition, plaque burden was also reduced in all treatment groups compared to the 9U group, but it was only significant in the 9AD group. This reduction was found in both the unstable (22.7 ± 18.0% versus 43.5 ± 12.8%, p = 0.02) and stable (28.9 ± 10.7% versus 47.2 ± 13.6%, p = 0.007) plaque regions (Figure 4). These results indicate that the combination of restored normal blood flow and atorvastatin promotes plaque regression.

### Decuffing restores artery lumen area, blood velocity, and wall shear stress

**Figure 5:**
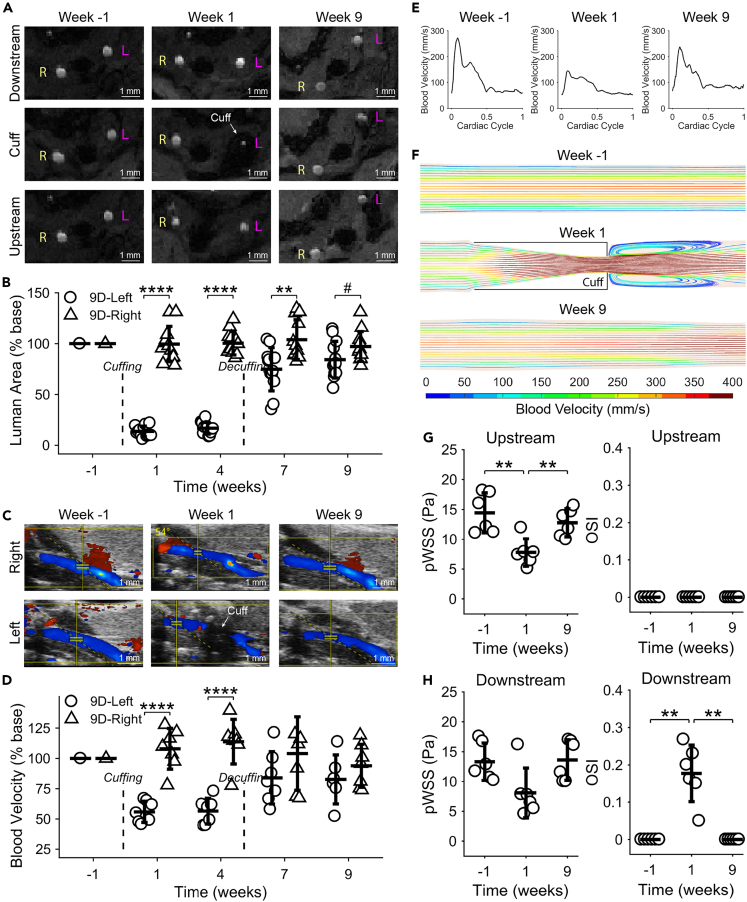
Decuffing restores normal carotid artery lumen patency, blood velocity, and wall shear stress Imaging and modeling data from 9-Decuffed (9D) mice. (A) Representative MRI slices of the downstream (stable plaque), cuff, and upstream (unstable plaque) regions of an instrumented carotid artery at −1 (baseline), 1 (cuffed), and 9 (decuffed) weeks after initial cuff placement. Scale bars are 1 mm. (B) Boxplot of lumen area as a percentage of baseline in the instrumented versus contralateral control arteries at −1, 1, 4, 7, and 9 weeks (n = 12 mice). See also Figure S1 and Table S5. (C) Representative ultrasound images of the instrumented and contralateral control arteries at −1, 1, and 9 weeks. Scale bars are 1 mm. (D) Boxplot of blood velocity as a percentage of baseline over time after initial cuff placement (n = 7 mice). (E) Representative blood velocity waveforms over the cardiac cycle (time is normalized) from one 9D mouse. (F) Representative CFD model of the instrumented carotid artery with blood velocity streamlines from one 9D mouse over time (blood flow is left to right). (G and H) Wall shear stress magnitude at peak systole (pWSS) and mean oscillatory shear index (OSI) in the (G) unstable and (H) stable plaque regions (n = 6 mice). (B, D, G, and H) Bars represent mean ± SD. ∗Indicates statistically significant difference for given comparison, wherein ^#^p<0.1, ∗∗p<0.01, and ∗∗∗∗p<0.0001. See also Table S4.

To demonstrate that decuffing restores normal blood flow, we evaluated lumen area at the point of maximum stenosis using MRI and inlet blood velocity using Doppler ultrasound for both the left (instrumented) and right (uninstrumented control) carotid arteries of decuffed mice (9D) at −1 (baseline), 1 (cuffed), 4 (cuffed), 7 (two weeks after decuffing), and 9 (four weeks after decuffing) weeks after initial cuff placement. These data are reported relative to baseline (i.e., before initial cuff placement). One week after initial cuff placement, the left carotid arteries of 9D mice exhibited a dramatic reduction in lumen area at the point of maximum stenosis compared to the control arteries (13.7 ± 5.2% versus 99.6 ± 17.4%, p<0.0001) (Figures 5A and 5B). This reduction was similar at four weeks (16.7 ± 5.8% versus 100.5 ± 11.6%, p<0.0001). At seven weeks (two weeks after decuffing), the maximum stenosis had dramatically improved (74.8 ± 21.2% versus 103.9 ± 19.5%, p = 0.004) and, at nine weeks (four weeks after decuffing), there was no significant difference between the left and right carotid arteries of the 9D mice (84.3 ± 18.1% versus 97.3 ± 14.6%, p = 0.07). We also evaluated lumen area restoration in 9D versus 9U mice (Figure S1 and Table S5). No statistical differences were seen between these groups at one and four weeks after cuffing, but at seven and nine weeks, as lumen area was restored in the 9D mice because of decuffing, both time points were significantly different between the two groups (74.8 ± 21.2% versus 20.1 ± 10.2% (p<0.0001) and 84.3 ± 18.1% versus 21.0 ± 9.6% (p<0.0001) at 7 and 9 weeks, respectively).

Peak systolic blood velocity at the inlet of each carotid artery of the 9D mice from Doppler ultrasound showed a nearly identical trend to the MRI data. The left carotid artery exhibited a dramatic reduction in blood velocity compared to the control artery at both one (55.9 ± 8.8% versus 107.9 ± 16.8%, p<0.0001) and four weeks (56.5 ± 10.6% versus 113.8 ± 18.5%, p<0.0001) after initial cuff placement (Figures 5C and 5D). At seven weeks (two weeks after decuffing), the blood velocity had increased in the instrumented arteries and was no longer statistically different from the control arteries (84.0 ± 21.6% versus 104.0 ± 30.3%, p = 0.36). Similar blood velocities were seen at nine weeks (four weeks after decuffing) (82.6 ± 20.2% versus 93.9 ± 17.7%, p = 0.31).

Finally, because WSS is the mechanical metric of flow that is most often associated with atherosclerosis, we also performed CFD of the instrumented carotid arteries of 9D mice at −1, 1, and 9 weeks after initial cuff placement to characterize the degree of WSS restoration that results from decuffing. Simulations were performed in a mouse-specific manner using the vessel geometry from MRI and inlet blood velocity over the cardiac cycle from ultrasound (Figure 5E) for a given mouse at each time point with the same mice followed over time. This approach provided a complete picture of the effects of decuffing on WSS by evaluating the same arteries at baseline (week −1), after cuff placement (week 1), and then four weeks after decuffing when lumen area (MRI) and blood velocity (ultrasound) were maximally restored (week 9). We evaluated two WSS metrics, WSS at peak systole (pWSS) to quantify magnitude and oscillatory shear index (OSI) to quantify multidirectionality over the cardiac cycle.

Importantly, our CFD models predicted trends over time that were consistent with the MRI and ultrasound data (Figure 5F). In the upstream region, pWSS was significantly reduced by cuff placement (week 1) compared to both baseline at week −1 (7.8 ± 2.3 Pa versus 14.4 ± 3.3 Pa, p = 0.009) and decuffed at week 9 (7.8 ± 2.3 Pa versus 12.8 ± 2.3 Pa, p = 0.009) (Figure 5G). In addition, there was no difference between the baseline and decuffed time points (p = 0.37), demonstrating that WSS magnitude was restored (the upstream segment does not experience multidirectional flow, so OSI was zero across all time points). Similar pWSS results were seen in the downstream region, though values did not reach significance between any of the time points, including cuffed (week 1) versus either baseline (week −1) or decuffed (week 9) because of an outlier at week 1 caused by high flow reversal (Figure 5H). However, mean OSI in the downstream region at week 1 (0.18 ± 0.08) was significantly higher compared to baseline (0.00 ± 0.00, p = 0.003) and week 9 (0.00 ± 0.00, p = 0.003). Also, there was no difference in OSI between the baseline and decuffed time points (p = 0.69). Thus, in both the upstream and downstream segments, deviations in WSS magnitude and directionality caused by placement of the blood flow-modifying cuff were resolved after decuffing.

## Discussion

Decades of research has established that the presence of disturbed blood flow within local arterial segments is a key aspect of the initiation and progression of atherosclerotic plaques in humans^2^^,^^3^^,^^7^^,^^8^^,^^12^^,^^17^^,^^18^^,^^19^ and animal models.^1^^,^^9^^,^^10^^,^^13^^,^^14^^,^^20^^,^^21^ Yet, a complete understanding of the relationship between blood flow and atherosclerosis also requires evaluation of the effects of normal flow on plaque fate. Herein, we filled this knowledge gap by demonstrating for the first time a causal relationship between restoration of normal blood flow within atherosclerotic arteries and plaque stabilization. A blood flow-modifying cuff was used to induce atherosclerosis in an artery that would otherwise remain non-diseased, whereas later decuffing allowed blood flow to return to normal. MR imaging, Doppler ultrasound, and CFD confirmed that decuffing allows recovery of lumen area, blood velocity, and WSS to near baseline values. Despite some variability in these mechanical data over the rather short four-week therapeutic window employed in this study, we consistently observed that, compared to untreated mice (5U and 9U), plaques in the decuffed mice had higher collagen in both the unstable (+144% and +89%, respectively) and stable (+83% and +78%) regions, lower lipid (−46% and −72%) in the unstable region, and lower macrophages (−64% and −72%) in the unstable region. This dramatic modification in plaque composition indicates that even partial restoration of normal blood flow causes plaque stabilization.

It is remarkable that the degree of plaque stabilization in mice treated with restored normal blood flow was comparable to those treated with atorvastatin, given the well-documented efficacy of the latter as a first-line therapeutic for atherosclerosis in patients that reduces the 10-year incidence of heart attack by ∼23%.^22^ Another interesting parallel is that statins induce this beneficial effect primarily by promoting plaque stabilization. For example, the EASY-FIT (Effect of AtorvaStatin therapY on FIbrous cap Thickness in coronary atherosclerotic plaque) trial with 70 patients found a significant increase in fibrous cap thickness (+69%), decrease in lipid arc (−27%), and decrease in macrophage grade (i.e., accumulation) (−38%) with 12 months of moderate atorvastatin therapy (20 mg/day).^23^ Similarly, another trial with 44 patients found a significant decrease in lipid-core burden index (−23%) with only 7 weeks of high-dose rosuvastatin (40 mg/day).^24^ Importantly, this study showed no change in plaque burden over this shorter timeframe. Although other clinical trials of statins over longer follow-up times (6–24 months) have shown decreases in plaque volume (typically, less than 10%),^25^ these changes are more modest compared to changes in plaque composition. These results closely align with ours herein for restoration of normal blood flow. Of interest, we also found that the combination of atorvastatin and restored normal blood flow induced an additive beneficial effect that not only caused further reductions in lipid and increases in collagen, but also caused a significantly lower plaque burden compared to the untreated mice at 9 weeks (−48% and −39% for unstable and stable plaque regions, respectively). This result indicates that the combined therapy promotes both plaque stabilization and regression. It also suggests that the therapeutic effects of statins and normal blood flow operate through different signaling pathways, which aligns with previous work demonstrating that statins and blood flow differentially regulate many endothelial genes.^26^^,^^27^

Although our study appears to be the first to directly consider the therapeutic effects of normal blood flow on atherosclerosis, previous studies provide indirect evidence of its beneficial effects. One clinical trial evaluating the association between disturbed flow and atherosclerosis in 374 patients demonstrated that, whereas the presence of low WSS in arterial segments at baseline was associated with decreased lumen area and increased plaque burden at 6 months follow-up, moderate and high WSS were associated with increased lumen area and decreased plaque burden.^7^ Another patient study showed similar results.^8^ In addition, studies of exercise, which increases blood flow, have also shown beneficial effects on atherosclerosis.^28^ Studies in ApoE^−/−^ mice have demonstrated that exercise promotes the formation of more stable plaques compared to sedentary controls with similar compositional changes to those reported herein, including higher collagen content.^29^^,^^30^ In humans, it is well accepted that regular exercise reduces the risk of cardiovascular events, likely by promoting more stable atherosclerotic plaque phenotypes.^31^

In conclusion, blood flow is a key regulator of the atherosclerotic state of an artery. It is well established that disturbed blood flow promotes plaque initiation and progression, whereas normal blood flow is protective. Herein, we demonstrated that restoration of normal blood flow in atherosclerotic arteries is also therapeutic by promoting plaque stabilization. These findings motivate the development of new therapeutic approaches that leverage the mechanosensitive nature of atherosclerosis, either through direct biomechanical modulation of the atherosclerotic artery or the administration of pharmaceuticals. Our results suggest that such a therapeutic could provide an additive beneficial effect when administered in parallel to traditional pharmacologic strategies such as statins.

### Limitations of the study

There are five primary limitations of our study to consider addressing in future work. First, we only used female ApoE^−/−^ mice. Because this study is the first to evaluate the therapeutic effects of a beneficial mechanical stimulus in the context of any vascular disease, we sought to minimize the potential of biological variables impacting our ability to observe differences between the treated and untreated groups. However, previous work has shown that the size and distribution of atherosclerotic plaques is similar between female and male ApoE^−/−^ mice,^32^^,^^33^ suggesting that plaques in male mice would respond similar to the treatment regimens used herein. Second, we assessed our therapeutics at an intermediate stage of plaque development. This approach limited inward growth of the plaques and their impact on blood flow because the plaques are smaller and arteries undergo outward remodeling to compensate for plaque development (up to ∼40% of plaque burden).^34^^,^^35^ Because plaques exist over a range of phenotypes that may influence their response to therapeutics, future work is needed to assess the effects of normal blood flow on more advanced plaques. Third, we applied our therapeutics over a relatively short timeframe of four weeks. Because the MRI data demonstrated improved lumen recovery over time, the beneficial effects stemming from restored normal blood flow were likely not established for that entire period. Nevertheless, we still observed highly significant improvements in plaque composition toward a more stable phenotype in mice treated with restored normal blood flow, suggesting that a longer timeframe of treatment would only lead to even better outcomes. Future work focusing on larger and more advanced plaque phenotypes should particularly consider longer time frames. Fourth, the CFD models did not consider artery curvature. CFD simulations with curvature at baseline and 9 weeks showed negligible differences for the readouts reported herein compared to the straightened models (Figure S2). Future work seeking to evaluate local shear and histological features (versus the current approach of averaging over the whole vessel segments) may need to include this complexity. Fifth, we did not directly correlate shear metrics to histology. Although many of the 9D (and 9U) mice used for histology were also imaged with MRI, Doppler ultrasound and CFD were performed late in the study with different mice (MRI was performed in these mice as well) for the sole purpose of supporting the MRI data in demonstrating that decuffing restores normal blood flow. However, directly correlating local shear metrics to features of the local plaque environment may require a more sophisticated approach, such as placement of fiducial markers on the vessels before collection for histology, the use of higher resolution imaging, and fluid-structure interaction modeling for more realistic boundary conditions. Thus, there are many technical challenges to consider when performing such correlation analyses that warrant additional studies.

## STAR★Methods

### Key resources table

**Table:** 

REAGENT or RESOURCE	SOURCE	IDENTIFIER
**Experimental models: Organisms/strains**		

Mouse: B6.129P2-Apoe^tm1Unc^/J	The Jackson Laboratory	Cat# 002052; RRID IMSR_JAX:002052:
Rodent atherogenic diet	Envigo (Teklad)	Cat#TD.88137

**Antibodies**		

Anti-mouse CD68	BioLegend	Cat# 137002; RRID: AB_2044004
Anti-rat IgG H&L (Alexa Fluor 647)	Abcam	Cat#ab150167; RRID: AB_2864291

**Chemicals, peptides, and recombinant proteins**		

Oil-Red-O	Electron Microscopy Sciences	Cat#26502-01
37% Formaldehyde	Fisher Scientific	Cat#BP531-500
Propylene Glycol	Sigma-Aldrich	Cat#398039-2L
Hematoxylin	Sigma-Aldrich	Cat#MHS80-2.5L
Ammonium Hydroxide	Sigma-Aldrich	Cat#221218-1L-A
Aqua PolyMount	Polysciences Inc.	Cat#18606-20
Picric Acid Solution	Sigma-Aldrich	Cat#197378-100G
Direct Red 80	Sigma-Aldrich	Cat#365548-5G
Fast Green FCF	Sigma-Aldrich	Cat#F7258-25G
Tissue-Tek Mounting Medium	Electron Microscopy Sciences	Cat#62552-01
PBS Powdered Solution	Fisher Scientific	Cat#BP655-1
4% Paraformaldehyde	Thermo Scientific	Cat#J19943-K2
30% Hydrogen Peroxide	Millipore Sigma	Cat#HX0635-3
Goat Serum	Abcam	Cat#ab7481
OCT Mounting Medium	Sakura	Cat#4583
Isopentane	Honeywell	Cat#M32631-1L
ORA-Plus	Perrigo	Cat# 0574-0303-16
Atorvastatin Calcium Tablets (20 mg)	Apotex Corp.	Cat# 60505-2579-09
Prolong Gold antifade reagent	Invitrogen	Cat#P36930
DAPI	Abcam	Cat#ab228549
Hydrochloric Acid	Sigma-Aldrich	Cat#32031-500ML
Ethanol	Decon Laboratories Inc.	Cat#2701
Acetone	Fisher Scientific	Cat#A929-1

**Critical commercial assays**		

Reagent Piccolo® Lipid Panel Plus Quantitative Determination for Piccolo® Blood Chemistry Analyzer or Piccolo® Xpress™ Chemistry Analyzer 10 Disc / Box	McKesseon Medical-Surgical	Cat# 832685

**Deposited data**		

Histology section microscopy images and analysis	This paper	figshare: https://doi.org/10.6084/m9.figshare.21926049

**Software and algorithms**		

MATLAB R2017a	Mathworks	https://www.mathworks.com/products/matlab.html
ImageJ	Schneider et al.^36^	https://imagej.net/ij/index.html
MATLAB and ImageJ original codes	This paper	figshare: https://doi.org/10.6084/m9.figshare.21926307
The Vascular Modeling Toolkit (VMTK) v1.6.1	OROBIX	http://www.vmtk.org/
Itk-SNAP v3.6	Yushkevichet al.^37^	http://www.itksnap.org/pmwiki/pmwiki.php?n=Main.HomePage
Vevo Lab v5.5.1	Visual Sonics	https://www.visualsonics.com/product/software/vevo-lab
Abaqus v6.14	Dessault Systemes	https://www.3ds.com/products-services/simulia/products/abaqus/
TetGen tetrahedral mesh generator	The Weierstrass Institute and Si^38^	https://wias-berlin.de/software/index.jsp?id=TetGen&lang=1

**Other**		

Axio Observer 5 inverted light microscope	Zeiss	https://www.micro-shop.zeiss.com/en/us/system/axio+observer-axio+observer+5-inverted+microscopes/10302/
LSM 800 confocal laser scanning microscope	Zeiss	https://www.zeiss.com/microscopy/en/products/light-microscopes/confocal-microscopes.html

### Resource availability

#### Lead contact

Further information and requests for resources and reagents should be directed to and will be fulfilled by the lead contact, Ryan M. Pedrigi (rpedrigi@unl.edu).

#### Materials availability

This study did not generate any new materials.

### Experimental model and subject details

This study was carried out in strict accordance with the recommendations in the Guide for the Care and Use of Laboratory Animals of the National Institutes of Health. The protocol was approved by the Institutional Animal Care and Use Committee of the University of Nebraska-Lincoln (Project ID: 2007). A total of 75 female ApoE^-/-^ mice on a C57BL/6J background were acquired at 11 weeks of age (Jackson Labs, strain#002052) and maintained in cages with bedding and *ad libitum* food and water in an environmentally-controlled animal facility. Mice were immediately placed on an atherogenic diet (Teklad, Envigo, TD.88137) that was maintained for the entirety of the experiments. Two weeks later (denoted week 0), all mice were instrumented with a blood flow-modifying cuff (Promolding) around the left common carotid artery and the contralateral carotid artery served as a control.^13^ Surgeries were performed under isoflurane gas anesthesia (4–5% induction and 2–3% for maintenance) and sustained-release buprenorphine (1 mg/kg via subcutaneous injection) was given at the time of surgery to provide analgesia for 72 h. Both atorvastatin groups received 0.22 mg (∼10 mg/kg) in an ORA-plus suspension vehicle by oral gavage daily from week 5 until the end of the experiments at week 9. In line with previous work,^30^^,^^39^ we validated that the atherogenic diet produced hypercholesterolemia and atorvastatin reduced total cholesterol levels (Figure S3 and Table S6). Mice from all groups were humanely sacrificed at week 9, except the 5U group, which was humanely sacrificed at week 5.

### Method details

#### Histology

Atherosclerotic plaque burden and constituents were assessed by histological processing of both carotid arteries from all mice. Mice were perfusion-fixed using 4% paraformaldehyde (Fisher Scientific) in PBS at mean arterial pressure. The carotid arteries, including the upper aortic arch and carotid bifurcations, were then extracted, embedded in OCT medium (Sakura), and snap frozen in a mixture of dry ice and isopentane (Sigma). The tissue block was cryosectioned at 8 μm thickness (Leica 1900CM) and serially collected from the innominate bifurcation of the right carotid artery to the bifurcation of both carotid arteries (thus, each cryosection contained one section from both the right and left carotid arteries). Sections were collected in a way to allow evaluation of multiple stains over the length of the arteries. This method provided an interval of 96 μm between sections for a given stain group.

Two basic stains were evaluated in this study. Oil red O (Sigma) staining was performed for evaluation of plaque burden and lipids. Picrosirius red (Sigma) staining was used for evaluation of collagen. Plaque burden was computed as the percentage of plaque area to external elastic lamina area.^40^ Basic stains were imaged with a Zeiss Axio Observer 5 microscope at 10X magnification using brightfield and polarized light, respectively. Stained regions of the oil red O and collagen section images were identified using a color threshold in ImageJ to render the images binary (for the collagen, two thresholds were used, one for red/yellow and the other for blue/green, and the results were summed). The threshold for each stain group of each mouse was the average identified by two independent observers who were masked to the identity of the experimental group. The identified threshold was held constant for all images of a stain group in each mouse.

CD68 immunofluorescence staining was also performed to identify macrophages. The sectioned tissue was fixed in −20°C acetone for 10 min prior to staining. It was then incubated at room temperature with a rat polyclonal anti-CD68 primary antibody (BioLegend) in 10% goat serum (1:250) for 1 hour. The tissue was then washed with PBS and incubated at room temperature in the dark with goat polyclonal anti-rat preadsorbed secondary antibody conjugated to Alexa Fluor 647 (Abcam) in 10% goat serum (1:250) for 1 hour. The sections were then counterstained with DAPI (0.0025%, Abcam) and embedded in Prolong Gold Antifade Mountant (Thermo Fisher) with a coverslip. Imaging was performed on a Zeiss LSM 800 confocal microscope using a 2x2 tile scan at 20x magnification with excitation/emission wavelengths of 650/651-800 nm. All confocal parameters were held constant across all sections of all mice. The fluorescence images were then converted to greyscale.

Quantification of all stains was done using a custom MATLAB program, as previously described.^15^ Briefly, each histological section was manually segmented using this program to identify the lumen, internal elastic lamina (IEL), and external elastic lamina (EEL) (for fluorescence images, a phase image was taken for each section that was used for segmentation). Once manually segmented, the program automatically identified the intima based on deviations between the lumen and internal elastic lamina contours (intima area was zero for non-diseased arterial sections). The binary (0 or 1) or greyscale (0 to 105—the maximum of the greyscale-converted images from the pink CD68 stain) pixel values within the intima were then summed and normalized by the total number of pixels in the intima. For the greyscale (CD68) stain, this calculation gave stain *intensity* per intima area, not stain area per intima area. To provide a better estimate of the latter, pixel values for all histological images of all mice were additionally normalized by 30 based on the mean threshold manually found to best identify the CD68 stain within the greyscale histological sections of representative mice. Thus, all stains are reported as stain area to intima area. Since sections were serially collected, all viable sections from the middle of each plaque in each arterial segment immediately upstream and downstream of the cuff within each mouse were included in the quantitative analysis for each stain. Sections were excluded if they were missing, damaged, or at the ends of the plaque where clear tapering was evident. Viable sections were identified by two independent observers in a blinded manner. The mean percentage of stain area to intima area was typically obtained from 3-4 sections and used as the value for a given stain/arterial segment/mouse (represented as a single data point on the given plot).

#### Magnetic resonance imaging

Lumen patency of the instrumented carotid arteries was evaluated in mice from the 9U and 9D groups using serial MRI at −1 (before cuff placement), 1, 4, 7, and 9 weeks after initial cuff placement (the same mice were followed over all time points). Prior to imaging, mice were anesthetized using 2% isoflurane gas and affixed in a cylindrical animal holder to maintain head and body position during imaging. Breathing rates were monitored by a pressure-based sensor (SA Instruments), maintaining 50 to 80 breaths per minute over the course of the imaging sequence. The carotid arteries were imaged over 14 slices with 0.5 mm thickness per slice from the carotid bifurcation towards the aortic arch using a 9.4 T (400 MHz) 89 mm vertical bore Varian magnet with a 4 cm millipede RF imaging probe with triple axis gradients (100 G/cm max). Images were collected as a gradient echo sequence (GEMS) with FOV of 23 × 23 mm and 256x256 matrix for an in-plane resolution of 90 μm with a TR of 120 ms, TE of 4.29 ms and four averages. The left and right carotid arteries were both segmented from the MRI slices using ITK-SNAP and then imported into ImageJ to measure the lumen cross-sectional area and Feret’s diameter of each carotid artery over the 14 slices (the average of the min and max Feret’s diameter was taken as the lumen diameter). Lumen area of the instrumented arteries at the point of maximum stenosis are reported as a percentage of lumen area prior to cuff placement (i.e., week −1).

#### Ultrasound

Blood velocity at the inlet of the carotid arteries was evaluated in a subset of the 9D mice used for MRI at the same weekly time points using the Vevo3100 (Fujifilm VisualSonics, Toronto, Canada) ultrasound system. An MX550D transducer (40 MHz center frequency) was used for Doppler Ultrasound imaging. Mice were anesthetized using 1-2% isoflurane with balance 100% O_2_ and placed on a heated stage (37°C) in the supine position. Hair on the neck region was removed (Nair) and ultrasound gel was applied liberally to the region. The transducer was placed in the sagittal plane along the carotid artery. Pulsed-wave Doppler measured blood velocity at the center of each artery over the cardiac cycle about 3 mm from the aortic bifurcation on both the left and right carotid arteries. Analysis of the data was done using Vevo Lab (version 5.6.1) offline. For comparisons over time, the average blood velocity at peak systole over three cardiac cycles for each mouse is reported relative to baseline (i.e., week -1) at each time point. For CFD analysis, the blood velocity averaged over three cardiac cycles was used for the inlet boundary condition for each mouse at each time point (Figure S4).

#### Computational fluid dynamics

Mouse-specific CFD simulations were created using the MRI (vessel geometry) and Doppler ultrasound (inlet blood velocity) measurements to determine wall shear stress in the instrumented artery at −1, 1, and 9 weeks after initial cuff placement (the same mice were followed over time). To reconstruct the artery geometry, a custom MATLAB code was used to construct a point cloud of the artery by placing the best-fit lumen diameters from MRI on a straightened vessel centerline, thus neglecting the small curvature of the arteries (Figure S5). This simplification had a negligible effect on the WSS readouts reported herein (Figure S2). Flow extensions were added to the inlet and outlet at a length of 1.5- and 7-fold of each diameter, respectively, following previous work.^8^^,^^12^^,^^14^ The surface of the artery was then converted into an STL file and the fluid domain was fully meshed with tetrahedral elements using TetGen (WIAS). We focused our analysis on the segments of the instrumented artery over the first 2 mm immediately upstream and downstream of the cuff because this is where plaque formation occurs (at the time prior to cuff placement or after decuffing when the exact location of the previous cuff placement could not be determined, the central portion of the artery was used). Here, a maximum element side length of 0.013 mm was assigned. The portions of the artery beyond these regions were assigned slightly courser mesh densities based on maximum element side lengths of 0.025 mm (2–3 mm away from the cuff) and 0.05 mm for the rest of the artery. This approach resulted in a total mesh density of approximately 2M elements for the cuffed arteries (week 1) and 2.5M elements for the baseline (week -1) and decuffed (week 9) arteries. The final mesh density was determined using a convergence test with a criteria of <2% in time-averaged wall shear stress (TAWSS).

Each meshed artery was imported into Abaqus to perform CFD on a computing cluster in the Holland Computing Center at the University of Nebraska using 256 cores per simulation. CFD models were setup using the same general approach as our previous work.^14^ Briefly, blood was assumed to be an incompressible Newtonian fluid with a viscosity of 3.5 mPa.s and density of 1050 kg/m^3^. The inlet of each artery was prescribed the mean pulsatile blood velocity waveform over the cardiac cycle obtained from Doppler ultrasound for each mouse at each time point and we assumed a plug profile over the vessel cross-section. The outlet was prescribed a pressure of zero and the artery wall was prescribed a no-slip boundary condition. Each simulation was run over three cardiac cycles to ensure fully-developed flow. We observed that shear stress values between the second and third cardiac cycles changed less than 2%, demonstrating convergence. A WSS vector was obtained for each element of the mesh at the artery wall interface from the third cardiac cycle and imported into a custom MATLAB program to quantify the magnitude of WSS at peak systole (pWSS), TAWSS, and oscillatory shear index (OSI).^1^^,^^14^ Mean values of pWSS and mean non-zero values of OSI are reported over the 2 mm artery segments immediately upstream and downstream of the cuff (OSI was averaged over elements containing non-zero values—greater than or equal to 0.01—within the analysis regions because OSI localizes to particular portions of recirculation zones; when no value of OSI was greater than or equal to 0.01, the mean was taken over the entire artery segment).

### Quantification and statistical analysis

All statistical tests were performed in Minitab. Quantities are reported as mean ± standard deviation (SD). For unpaired comparisons, if the data were normally distributed (determined by a Shapiro-Wilk test), group comparisons were performed using a Welch’s one-way analysis of variance (ANOVA) and pairwise comparisons, including those post-hoc of the ANOVA, were performed using an unpaired *t* test with equal or unequal variances based on an F-test. Otherwise, a Kruskal-Wallis test (group comparisons) and Mann Whitney U test (pairwise comparisons) with Levene’s test for variances were used. For paired comparisons, a repeated-measures ANOVA for group comparisons and paired *t*-test for individual comparisons were used for normally distributed data, otherwise a Friedman test and Wilcoxon signed-rank test were used. Pairwise comparisons were done using a one-tailed approach when the hypothesized effect was direction dependent or a two-tailed approach when it was not. Multiple comparisons were corrected using the Holm-Bonferroni method. An adjusted p value of less than 0.05 was considered statistically significant.

## Data Availability

• Histology data, including raw microscopy images, binarized images of the basic stains, and MATLAB quantification, have been deposited at Figshare and are publicly available as of the date of publication. The DOI is listed in the key resources table. Medical imaging and computational model data reported in this paper will be shared by the lead contact upon request. • All original code used to analyze the histology data has been deposited at Figshare and is publicly available as of the date of publication. The DOI is listed in the key resources table. • Any additional information necessary for reanalysis of the data reported in this paper is available upon request from the lead contact.
