# Supplementary material for: Restoration of normal blood flow in atherosclerotic arteries promotes plaque stabilization
Source: iScience. 2023 Apr 27;26(6):106760. doi: 10.1016/j.isci.2023.106760 (PMC10206490; doi:10.1016/j.isci.2023.106760)
Supplement: Document S1. Figures S1–S5 and Tables S1–S6 [file mmc1.pdf]

iScience, Volume *26*

Supplemental information

Restoration of normal blood flow

in atherosclerotic arteries

promotes plaque stabilization

Morgan A. Schake, Ian S. McCue, Evan T. Curtis, Thomas J. Ripperda Jr., Samuel Harvey, Bryan T. Hackfort, Anna Fitzwater, Yiannis S. Chatzizisis, Forrest M. Kievit, and Ryan M. Pedrigi

Supplemental Tables

Table S1. Results of all statistical comparisons for data in Figure 2 (histology of the unstable plaque region). The *p*-values were corrected for multiple comparisons using the Holm-Bonferroni method.

Figure 2B

	p-value
ANOVA	0.00069

	Comparison	Normality	Variance	Tails	p-value	Rank	Corr Factor	Adjusted p-value	Stars
1	5U v 9D	yes	equal	1	0.00807	5	5	0.04035	*
2	5U v 9A	yes	equal	1	0.06582	6	4	0.26328	-
3	5U v 9AD	yes	equal	1	0.00070	2	8	0.00560	**
4	9U v 9D	yes	unequal	1	0.00072	3	7	0.00507	**
5	9U v 9A	yes	unequal	1	0.00210	4	6	0.01258	*
6	9U v 9AD	yes	unequal	1	0.00047	1	9	0.00422	**
7	9D v 9A	yes	equal	2	0.46500	9	1	0.46500	-
8	9D v 9AD	yes	equal	1	0.35470	8	2	0.70939	-
9	9A v 9AD	yes	equal	1	0.12332	7	3	0.36997	-

Figure 2C

	p-value
ANOVA	0.00013

	Comparison	Normality	Variance	Tails	p-value	Rank	Corr Factor	Adjusted p-value	Stars
1	5U v 9D	yes	equal	1	0.00002	1	9	0.00020	***
2	5U v 9A	yes	equal	1	0.00265	4	6	0.01588	*
3	5U v 9AD	yes	equal	1	0.09246	6	4	0.36986	-
4	9U v 9D	yes	unequal	1	0.00016	2	8	0.00130	**
5	9U v 9A	yes	equal	1	0.00225	3	7	0.01576	*
6	9U v 9AD	yes	equal	1	0.01680	5	5	0.08399	-
7	9D v 9A	yes	equal	2	0.34600	7	3	1.00000	-
8	9D v 9AD	yes	equal	1	0.90753	9	1	0.90753	-
9	9A v 9AD	yes	equal	1	0.72180	8	2	1.00000	-

Figure 2D

	p-value
ANOVA	0.000012

	Comparison	Normality	Variance	Tails	p-value	Rank	Corr Factor	Adjusted p-value	Stars
1	5U v 9D	yes	equal	1	0.00053	3	7	0.00372	**
2	5U v 9A	yes	unequal	1	0.02983	7	3	0.08949	#
3	5U v 9AD	yes	equal	1	0.00000	1	9	0.00003	****
4	9U v 9D	yes	equal	1	0.00088	4	6	0.00528	**
5	9U v 9A	yes	unequal	1	0.06858	8	2	0.13715	-
6	9U v 9AD	yes	unequal	1	0.00002	2	8	0.00015	***
7	9D v 9A	yes	equal	2	0.64338	9	1	0.64338	-
8	9D v 9AD	yes	equal	1	0.00159	5	5	0.00793	**
9	9A v 9AD	yes	equal	1	0.00309	6	4	0.01236	*

Table S2. Results of all statistical comparisons for data in Figure 3 (histology of the stable plaque region). The *p*-values were corrected for multiple comparisons using the Holm-Bonferroni method.

Figure 3B

	p-value
**KW**	0.054

	Comparison	Normality	Variance	Tails	p-value	Rank	Corr Factor	Adjusted p-value	Stars
1	5U v 9D	no	equal	1	0.46329	8	2	0.92659	-
2	5U v 9A	no	equal	1	0.41210	7	3	1.00000	-
3	5U v 9AD	no	unequal	1	0.00805	1	9	0.07243	#
4	9U v 9D	yes	equal	1	0.17765	5	5	0.88825	-
5	9U v 9A	no	equal	1	0.29573	6	4	1.00000	-
6	9U v 9AD	yes	unequal	1	0.00880	2	8	0.07038	#
7	9D v 9A	no	equal	2	0.85633	9	1	0.85633	-
8	9D v 9AD	yes	unequal	1	0.01013	3	7	0.07088	#
9	9A v 9AD	no	unequal	1	0.02457	4	6	0.14742	-

Figure 3C

	p-value
**KW**	0.064

	Comparison	Normality	Variance	Tails	p-value	Rank	Corr Factor	Adjusted p-value	Stars
1	5U v 9D	yes	equal	1	0.05600	4	6	0.33600	-
2	5U v 9A	no	equal	1	0.18300	7	3	0.54900	-
3	5U v 9AD	no	equal	1	0.01400	2	8	0.11200	-
4	9U v 9D	yes	equal	1	0.04700	3	7	0.32900	-
5	9U v 9A	no	equal	1	0.06200	5	5	0.31000	-
6	9U v 9AD	no	equal	1	0.00890	1	9	0.08010	#
7	9D v 9A	no	equal	2	0.96629	9	1	0.96629	-
8	9D v 9AD	no	equal	1	0.23500	8	2	0.47000	-
9	9A v 9AD	no	equal	1	0.16600	6	4	0.66400	-

Figure 3D

	p-value
**ANOVA**	0.000054

	Comparison	Normality	Variance	Tails	p-value	Rank	Corr Factor	Adjusted p-value	Stars
1	5U v 9D	yes	equal	1	0.00240	3	7	0.01680	*
2	5U v 9A	yes	unequal	1	0.02209	7	3	0.06627	#
3	5U v 9AD	yes	equal	1	0.00001	2	8	0.00007	****
4	9U v 9D	yes	equal	1	0.00326	4	6	0.01956	*
5	9U v 9A	yes	equal	1	0.00769	6	4	0.03077	*
6	9U v 9AD	yes	equal	1	0.00000	1	9	0.00003	****
7	9D v 9A	yes	equal	2	0.55043	9	1	0.55043	-
8	9D v 9AD	yes	equal	1	0.00690	5	5	0.03450	*
9	9A v 9AD	yes	equal	1	0.11652	8	2	0.23303	-

Table S3. Results of all statistical comparisons for data in Figure 4 (plaque burden). The *p*-values were corrected for multiple comparisons using the Holm-Bonferroni method.

Figure 4A

	**p-value**
**ANOVA**	0.038

	Comparison	Normality	Variance	Tails	p-value	Rank	Corr Factor	Adjusted p-value	Stars
1	5U v 9D	yes	equal	1	0.33454	7	3	1.00000	-
2	5U v 9A	yes	equal	1	0.82908	9	1	0.82908	-
3	5U v 9AD	yes	equal	1	0.08698	3	7	0.60883	-
4	9U v 9D	yes	equal	1	0.01630	2	8	0.13036	-
5	9U v 9A	yes	equal	1	0.19625	5	5	0.98125	-
6	9U v 9AD	yes	equal	1	0.00228	1	9	0.02051	*
7	9D v 9A	yes	equal	2	0.29930	6	4	1.00000	-
8	9D v 9AD	yes	equal	1	0.42623	8	2	0.85246	-
9	9A v 9AD	yes	equal	1	0.09298	4	6	0.55786	-

Figure 4B

	**p-value**
**ANOVA**	0.035

	Comparison	Normality	Variance	Tails	p-value	Rank	Corr Factor	Adjusted p-value	Stars
1	5U v 9D	yes	equal	1	0.52810	7	3	1.00000	-
2	5U v 9A	yes	equal	1	0.54892	8	2	1.00000	-
3	5U v 9AD	yes	equal	1	0.05885	4	6	0.35309	-
4	9U v 9D	yes	equal	1	0.10035	6	4	0.40140	-
5	9U v 9A	yes	equal	1	0.09708	5	5	0.48541	-
6	9U v 9AD	yes	equal	1	0.00082	1	9	0.00737	**
7	9D v 9A	yes	equal	2	0.96177	9	1	0.96177	-
8	9D v 9AD	yes	equal	1	0.05585	3	7	0.39097	-
9	9A v 9AD	yes	equal	1	0.03435	2	8	0.27479	-

Table S4. Results of all statistical comparisons for data in Figure 5 (imaging and modeling of 9D mice). The *p*-values were corrected for multiple comparisons using the Holm-Bonferroni method.

Figure 5B

	Comparison	Normality	Variance	Tails	p-value	Rank	Corr Factor	Adjusted p-value	Stars
1	Week 1	yes	unequal	1	0.00000	2	3	0.00000	****
2	Week 4	yes	unequal	1	0.00000	1	4	0.00000	****
3	Week 7	no	equal	2	0.00200	3	2	0.00400	**
4	Week 9	yes	equal	2	0.06700	4	1	0.06700	#

Figure 5D

	Comparison	Normality	Variance	Tails	p-value	Rank	Corr Factor	Adjusted p-value	Stars
1	Week 1	yes	equal	1	0.00000	1	4	0.00002	****
2	Week 4	yes	equal	1	0.00001	2	3	0.00002	****
3	Week 7	yes	equal	2	0.17900	3	2	0.35800	-
4	Week 9	yes	equal	2	0.30500	4	1	0.30500	-

Figure 5G

WSS-Up	p-value
RM ANOVA	0.00300

	Comparison	Normality	Variance	Tails	p-value	Rank	Corr Factor	Adjusted p-value	Stars
1	-1 vs 1	yes	equal	1	0.00296	1	3	0.00888	**
2	-1 vs 9	yes	equal	2	0.37100	3	1	0.37100	-
3	1 vs 9	yes	equal	1	0.00430	2	2	0.00860	**

OSI-Up	p-value
Friedman	0.51300

Figure 5H

WSS-Down	p-value
Friedman	0.13500

OSI-Down	p-value
RM ANOVA	0.0002

	Comparison	Normality	Variance	Tails	p-value	Rank	Corr Factor	Adjusted p-value	Stars
1	-1 vs 1	yes	Unequal	1	0.00114	1	3	0.00342	**
2	-1 vs 9	yes	Unequal	2	0.69100	3	1	0.69100	-
3	1 vs 9	yes	Unequal	1	0.00114	1	3	0.00342	**

Table S5. Results of all statistical comparisons for data in Figure S1 (MR imaging of the 9D vs 9U groups), related to Figure 5. The *p*-values were corrected for multiple comparisons using the Holm-Bonferroni method.

Figure S1

	Comparison	Normality	Variance	Tails	p-value	Rank	Corr Factor	Adjusted p-value	Stars
1	Week 1	yes	equal	2	0.35900	3	2	0.71800	-
2	Week 4	yes	unequal	2	0.70900	4	1	0.70900	-
3	Week 7	yes	unequal	2	0.00000	2	3	0.00000	****
4	Week 9	yes	equal	2	0.00000	1	4	0.00000	****

Table S6. Results of all statistical comparisons for data related to STAR Methods and Figure S3 (cholesterol). The *p*-values were corrected for multiple comparisons using the Holm-Bonferroni method.

Figure S3

**Week -1**	**p-value**
**KW**	0.68300

**Week 4**	**p-value**
**KW**	0.28300

**Week 6**	**p-value**
**ANOVA**	0.00010

	**Comparison**	**Normality**	**Variance**	**Tails**	**p-value**	**Rank**	**Corr Factor**	**Adjusted p-value**	**Stars**
1	9U v 9D	yes	equal	2	0.78900	2	2	1.00000	-
2	9U v 9A	yes	equal	1	0.00016	3	1	0.00016	***
3	9D v 9A	yes	equal	1	0.00015	1	3	0.00045	***

**Week 9**	**p-value**
**ANOVA**	0.00012

	**Comparison**	**Normality**	**Variance**	**Tails**	**p-value**	**Rank**	**Corr Factor**	**Adjusted p-value**	**Stars**
1	9U v 9D	yes	equal	2	0.32500	3	1	0.32500	-
2	9U v 9A	yes	unequal	1	0.00017	1	3	0.00051	***
3	9D v 9A	yes	equal	1	0.00055	2	2	0.00110	**

Supplemental Figures

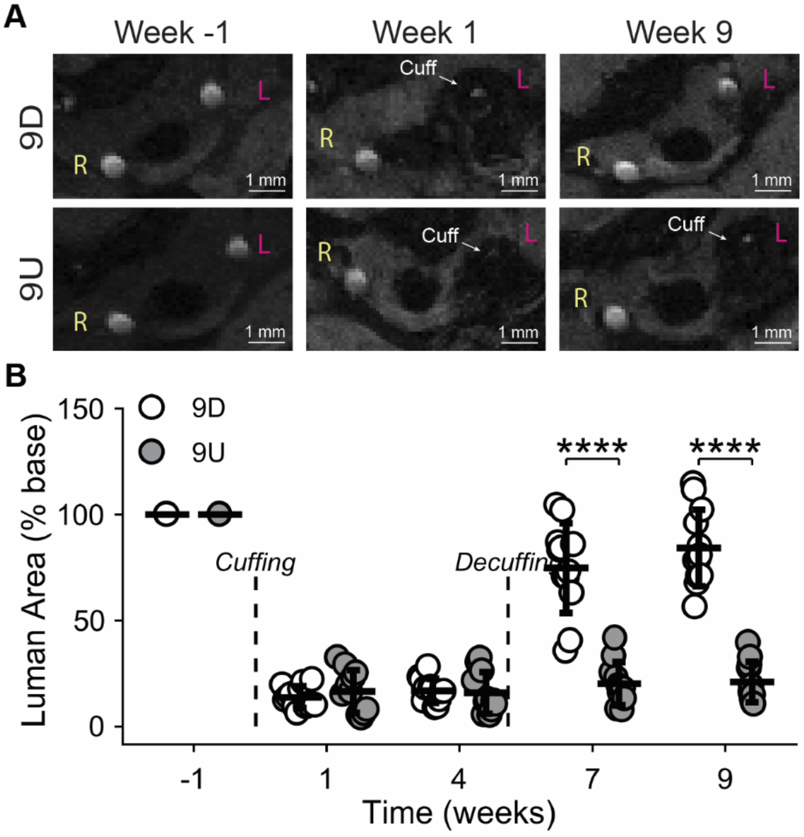

Figure S1. Decuffing restores carotid artery lumen patency compared to maintaining the cuff for the duration of the experiments, related to Figure 5. (A) Representative MRI slices within the cuff region (or center of the artery in the case of baseline) from the 9-Decuffed (9D) and 9-Untreated (9U) groups. Scale bars are 1 mm. (B) Box plot of lumen area as a percentage of baseline in the instrumented arteries from the 9D (*n*=12 mice) versus 9U (*n*=11 mice) groups at -1, 1, 4, 7, and 9 weeks after initial cuff placement. Bars represent mean±SD. *Indicates statistically significant difference for given comparison, wherein ****p<0.0001. See also **Table S5**.

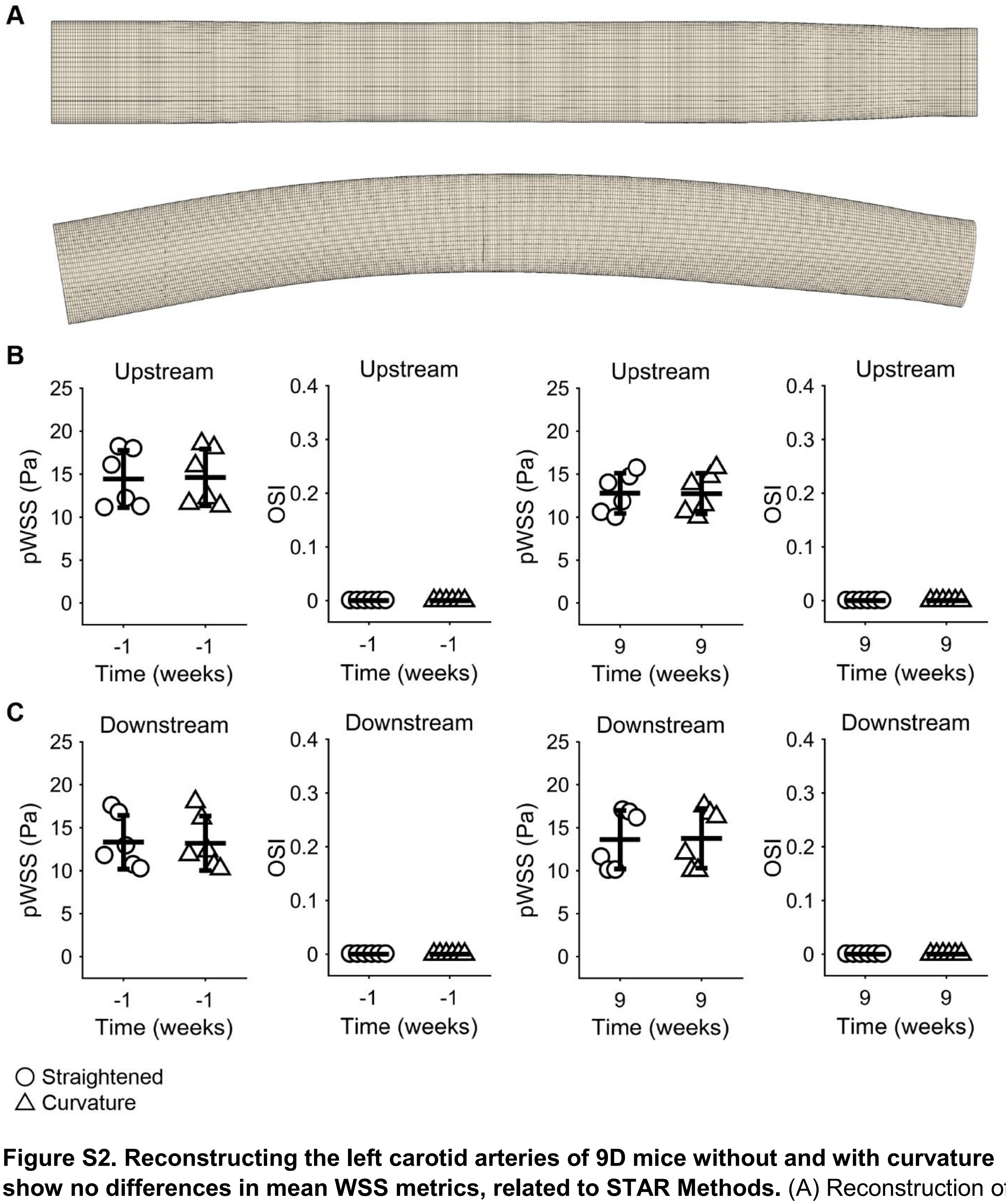

Figure S2. Reconstructing the left carotid arteries of 9D mice without and with curvature show no differences in mean WSS metrics, related to STAR Methods. (A) Reconstruction of a representative 9D mouse at 9 weeks without curvature (or straightened; top) versus with the natural curvature (bottom). (B-C) Plots of WSS at peak systole (pWSS) and OSI in the (B) upstream and (C) downstream vessel segments at weeks -1 and 9 (*n*=6 mice per group). Bars represent mean±SD. Statistical tests were not performed on these data.

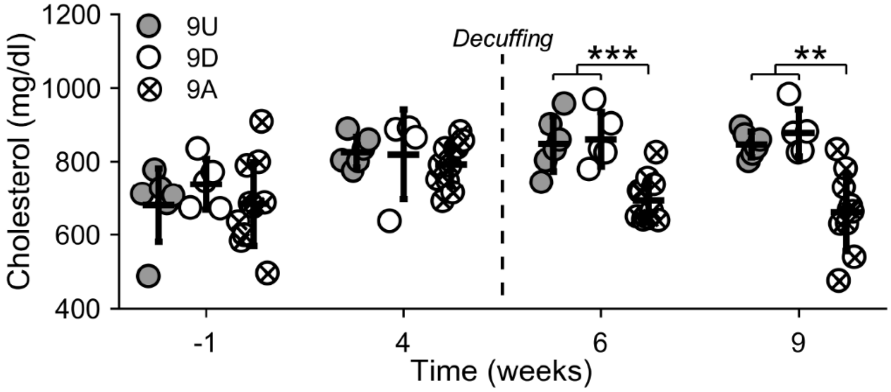

Figure S3. The Envigo (Teklad) atherogenic diet fed to ApoE$^{-/-}$ mice in this study induces severe hypercholesterolemia and atorvastatin reduces cholesterol levels, a validation related to STAR Methods. Total cholesterol (*n*=5-11 mice per group) was measured from a retro-orbital bleed after a 4 h fast using a Piccolo Xpress blood chemistry analyzer (Abbott) in 9-Untreated (9U), 9-Decuffed (9D), and 9-Atorvastatin (9A) mice over time. The same mice were followed over time. Bars represent mean±SD. *Indicates statistically significant difference for comparison given parenthetically, wherein ***p*<0.01 and ****p*<0.001. See also **Table S6**.

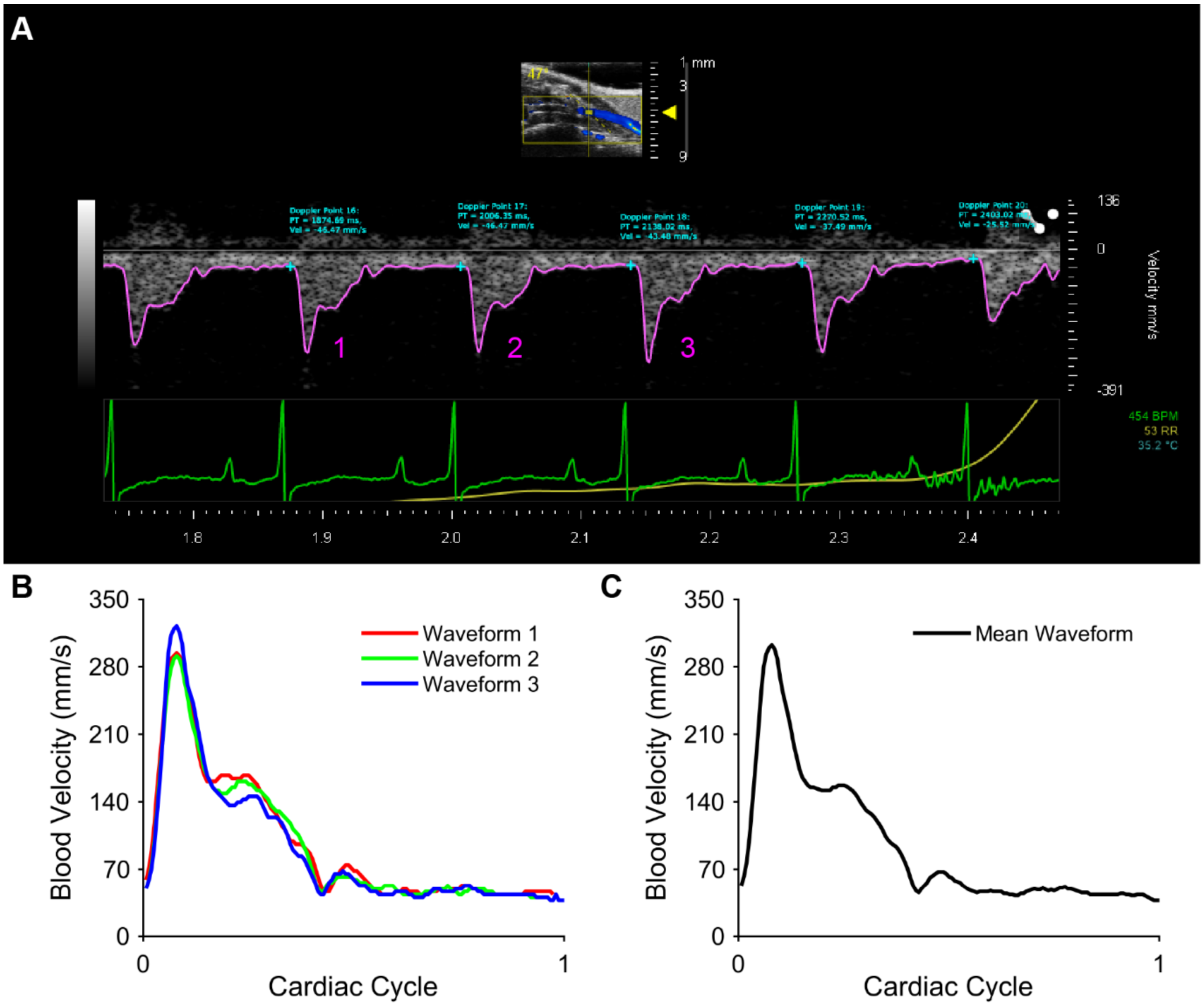

Figure S4. Workflow for creating inlet blood velocity waveforms from Doppler ultrasound for the CFD models, related to STAR Methods. (A) Raw blood velocity waveforms (pink lines) measured with Doppler ultrasound and heart rate (green line) from the left carotid artery of a representative mouse prior to cuff placement (week -1). (B) Plot of three waveforms extracted from the raw ultrasound data. (C) Mean of the three waveforms, which was used as the inlet boundary condition for the CFD model associated with this mouse and time point.

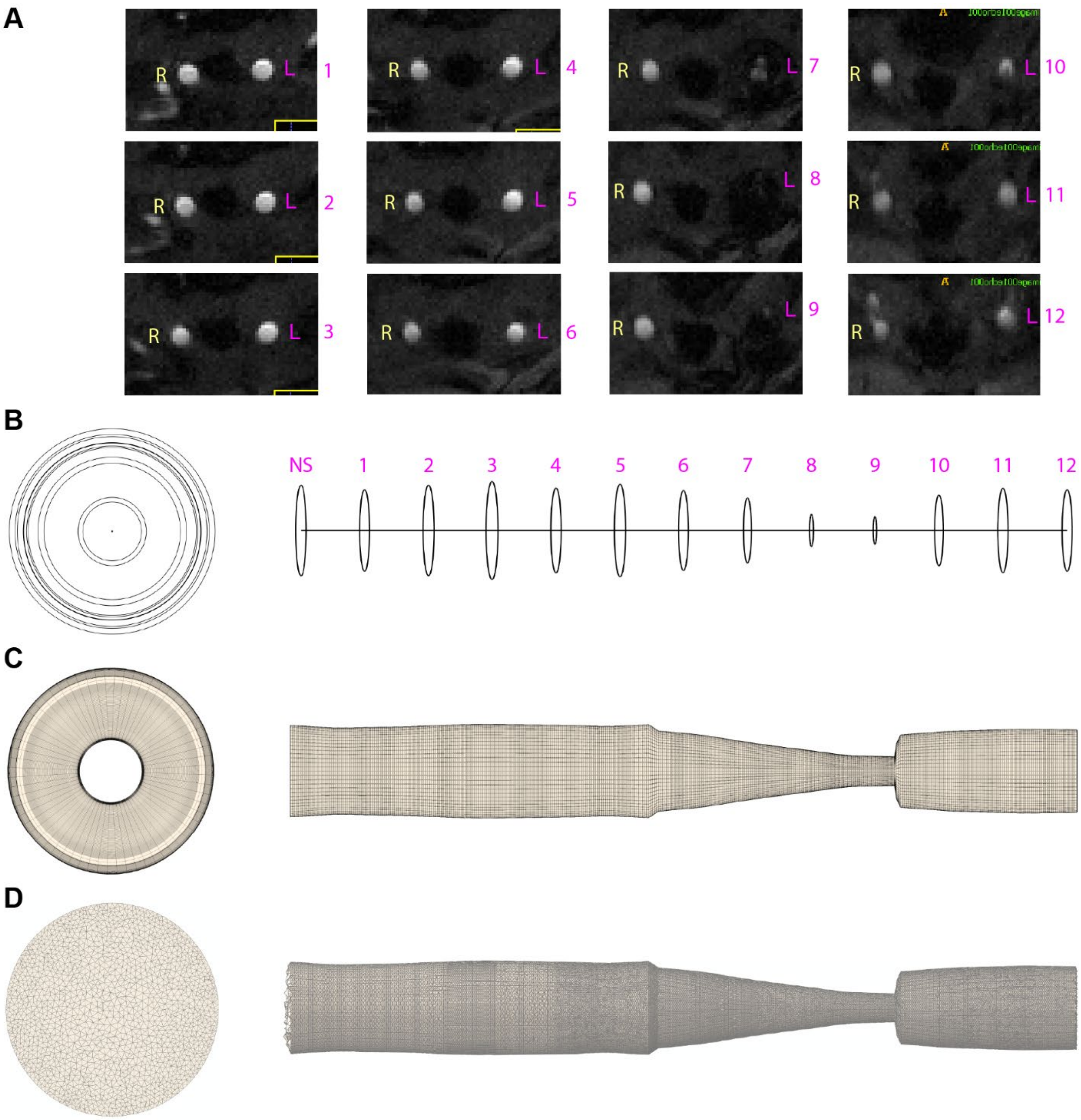

Figure S5. Workflow for developing CFD meshes of the carotid arteries from MRI, related to STAR Methods. (A) MRI slices through both carotid arteries of a representative mouse instrumented with a blood flow-modifying cuff around the left carotid artery (the first slice is not shown (NS) for clarity, but is similar to 1-5). The left (L) and right (R) arteries are identified in each image. (B-D) The cuffed artery reconstruction at three stages showing both cross-section and side views. (B) The best-fit diameter obtained from each MRI slice was placed along a straightened centerline (curvature of the arteries was neglected in our CFD models). (C) Each artery segment—upstream, cuff, and downstream—was smoothed and the vessel discretized using a user-defined number of nodes for export as a surface mesh. (D) The surface mesh was imported into TetGen and the fluid domain meshed using tetrahedral elements. Note: inlet and outlet extensions are not shown for clarity.
